# In Situ SEM Torsion Test of Metallic Glass Microwires Based on Micro Robotic Manipulation

**DOI:** 10.1155/2017/6215691

**Published:** 2017-08-23

**Authors:** Chenchen Jiang, Haojian Lu, Ke Cao, Wenfeng Wan, Yajing Shen, Yang Lu

**Affiliations:** ^1^Department of Mechanical and Biomedical Engineering, City University of Hong Kong, Kowloon, Hong Kong; ^2^Center for Advanced Structural Materials (CASM), Shenzhen Research Institute, City University of Hong Kong, Shenzhen 518057, China; ^3^Centre for Robotics and Automation, Shenzhen Research Institute, City University of Hong Kong, Shenzhen 518057, China

## Abstract

Microwires, such as metallic, semiconductor, and polymer microwires and carbon fibers, have stimulated great interest due to their importance in various structural and functional applications. Particularly, metallic glass (MG) microwires, because of their amorphous atoms arrangement, have some unique mechanical properties compared with traditional metals. Despite the fact that substantial research efforts have been made on the mechanical characterizations of metallic glass microwires under tension or flexural bending, the mechanical properties of microwires under torsional loading have not been well studied, mainly due to the experimental difficulties, such as the detection of torsion angle, quantitative measurement of the torsional load, and the alignment between the specimen and torque meter. In this work, we implemented the in situ SEM torsion tests of individual La_50_Al_30_Ni_20_ metallic glass (MG) microwires successfully based on a self-developed micro robotic mechanical testing system. Unprecedented details, such as the revolving vein-pattern along the torsion direction on MG microwires fracture surface, were revealed. Our platform could provide critical insights into understanding the deformation mechanisms of other microwires under torsional loading and can even be further used for robotic micromanufacturing.

## 1. Introduction

Microwires, such as metallic [1, 2], semiconducting [3, 4], and composite microwire [5, 6], biomaterial fiber [7], and carbon fiber [8, 9], have unusual mechanical and physical properties, making them promising for various mechatronic applications in micro electronics devices [10] or solar cells [11]. For example, polymer microwires with high elasticity even can function as a spring element to produce jumping or flapping motions in microrobots [12]. ZnO microwires, on the other hand, which have unique piezoelectric property, have been demonstrated to act as microsensor or field effect transistor [13]. Among those crystalline and noncrystalline microstructured materials, BMG (bulk metallic glass) has received tremendous research attention because of its unique physical and mechanical properties such as ultrahigh strength, high hardness, and large elastic strain [14, 15] due to the amorphous state of the atoms. Compared with the normal metals having crystalline lattice structures, which can facilitate dislocation movement under stress, making them soft and ductile, MGs, on the other hand, are normally hard and brittle at bulk scales [16]. Recently, MG microwires have received increased interests due to their different properties compared to their bulk counterparts; for instance, magnetic metallic glass microwires exhibit extremely soft magnetic behavior because of the absence of magnetocrystalline anisotropy, grain boundaries, and crystalline structure defects [17–20]. However, in-depth understanding of the mechanical properties of these novel MG micromaterials is still necessary for developing new applications, such as micro/nanoelectromechanical system (MEMS/NEMS) devices [21], heterogeneous catalysts [22], and magnetic sensors [23]. What is more, as the various microwires' applications circumstances have become complicated, the mechanical property of these materials has become a bottleneck constraint for long service time.

Although there have been extensive studies on the mechanical behavior of microwire materials in the past two decades, such as static tensile test [24–27], micro/nanoindentation measurement [28–30], bending measurement [31–33], and dynamic resonance frequency fatigue test [34, 35], little was reported on the behavior of microwire under the torsional loading [36–38]. Torsion of thin wires is a fundamental and excellent approach to explore the mechanical behavior, from elastic deformation, through yielding, to the strain-hardening regime. The reason for the rareness of torsion test of microwire was the great challenge involved in the experiment, such as the alignment between the specimen and the rotation axis, the detection of torsion angle, and the sensibility and calibration of torque meter. In this paper, we investigated the torsion fracture behavior of the La_50_Al_30_Ni_20_ MG microwire under in situ SEM and compared the fracture surface with tensile loading test [24–26] based on a self-developed micro robotic mechanical testing system. After analyzing the pattern on the fracture surface, the fracture mechanism of the microwire under torsion loading was proposed. The fracture resulted from the fact that the local temperature became very high to the melting point of the MG material and a fluid layer was generated; then the nucleated nano/microvoid caused the failure. The interesting revolved vein-pattern microstructures were firstly observed by the robot system we developed which we believe can be used in many other applications in the future, for example, micro assembly of nanoelectronic devices.

## 2. Sample and Experimental Procedures

### 2.1. MG Microwire Preparation

The metallic glass (MG) microwire samples (dia. 70 *μ*m) used in this work are fabricated by rapid quenching of alloy proportions from their liquid mixture. As the mechanical or magnetic property of the microwire is highly related to the microstructure of the materials [39, 40], the structure and composition should be confirmed before experiment. The chemical composition of the metallic glass is evaluated through the energy dispersive X-ray spectroscopy (EDS) studies carried out on the MG microwires, which reaffirmed the composition to be approximately La_50_Al_30_Ni_20_ (in atomic%). X-ray diffraction (XRD) studies on the MG microwires were carried out to confirm the amorphous nature of the material.

### 2.2. Micro Robotic Mechanical Testing System

**Figure 1:**
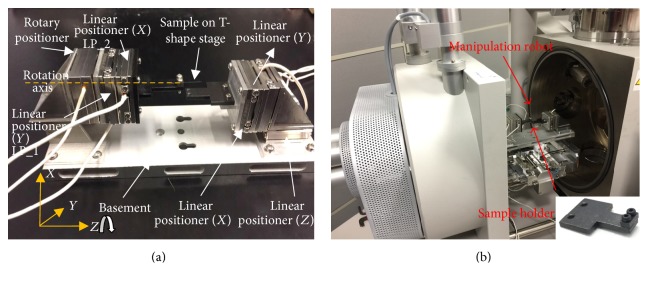
(a) is the photography of the robot and the illustration of the different key parts. The rotation axis is along the *Z* direction of the world coordinate. (b) shows that the small footprint of the robot is suitable for the in situ SEM experiment. The inset image is magnification of the T-shape stage with screws used to clamp the sample.

The self-developed micro robotic mechanical testing system is illustrated in Figure 1(a). The robot mainly comprised two parts [41–43]. The left motion part includes a rotary positioner and two linear positioners. If we set the world coordinate as Figure 1(a) shows, the rotation axis is along the *Z* direction. Upon the rotary positioner (RP), the linear positioner (LP_1) which moves in *Y* direction is joined. Then another linear positioner (LP_2) that moves in *X* direction is connected to the first one. LP_1 and LP_2's movement directions are mutually perpendicular. Each nanopositioner of the robot is responsible for one independent movement; thereby the left part of the robot has three degrees of freedom (DOFs) in total: two mutually perpendicular translational movements (along *X* and *Y* directions, resp.) and one rotation (the rotation axis is along *z*-axis). The right part includes three linear positioners, which can move independently in *X*, *Y*, *Z* directions, as Figure 1(a) shows. A metal basement is used to fix the two parts. Additionally, two T-shape stages were fabricated to clamp the sample at each side as the inset image shows. With the small footprint of the robot setup, it is suitable for SEM chamber for in situ experiment, as shown by Figure 1(b).

As to the parameters of the positioners, the travel range, resolution, and repeatability for the rotary positioner RP are 360° endless, (1 × 10^−6^)° and 5% over the full range, respectively. The travel range, resolution, and repeatability of the linear positioners are 20 mm, 1 nm, and 50 nm, respectively. Due to their high accuracy, the compact drive units can achieve the challenging positioning task of precise alignment in torsion test.

### 2.3. Experimental Setup

At first, the MG microwire sample was fixed between the T-shape stage on the left part and the metal plate by screwing. Then the robot was put in the SEM chamber and connected with the control box through the port. Because the SEM imaging system can only provide the 2D image information, it is very difficult to obtain the position of the sample directly based on the SEM images. An automatic forward-backward alignment strategy was proposed to address this challenge.

**Figure 2:**
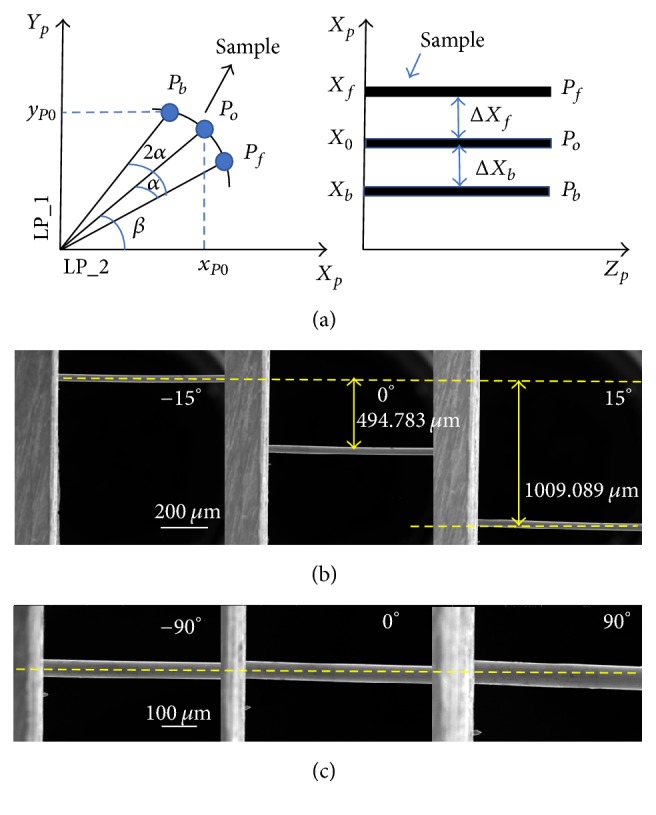
(a) Illustration of the alignment process. (b) The images captured from SEM at different angles can be used to calculate how much LP_1 and LP_2 have to move. (c) The alignment result shows that no matter how many rotations there are, the sample remains almost at the same position.

As shown in Figure 2(a), first microscope image is captured. Then rotate rotary positioner by *α* degrees so that the second microscope image can be captured. After that, rotate rotary positioner by 2*α* degrees so that the third microscope image can be captured. After these procedures, all the information for the sample alignment strategy has been obtained. Simplified coordinate diagram shows the calculation process of the proposed alignment principle. The detailed alignment strategy is illustrated in our previous work [44, 45]. After calculation, the movement of LP_1 and LP_2 is given as follows:

(1) xo=Δxp+Δxn2cos⁡α−1yo=Δxp−Δxn2sin⁡α.

Before the sample alignment, when the micro robotic mechanical testing system rotates with angles −15°, 0°, and +15°, as shown in Figure 2(b), the maximum position difference between the three images is 1009.089 *μ*m. After the sample alignment, when the micro robotic mechanical testing system rotates with angles −90°, 0°, and +90°, as depicted in Figure 2(c), the sample almost remains at the same position.

After aligning the sample along the axis of the rotation positioner, we control the T-shape stage on the right part of the robot to approach the freestanding side of the sample slowly. We can set the gap between the two stages as needed by using linear positioner at *Z* direction. Then we open the SEM chamber and fix the sample on the right T-shape stage also by screwing a metal plate for subsequent in situ SEM testing.

### 2.4. Torsion Process inside SEM

**Figure 3:**
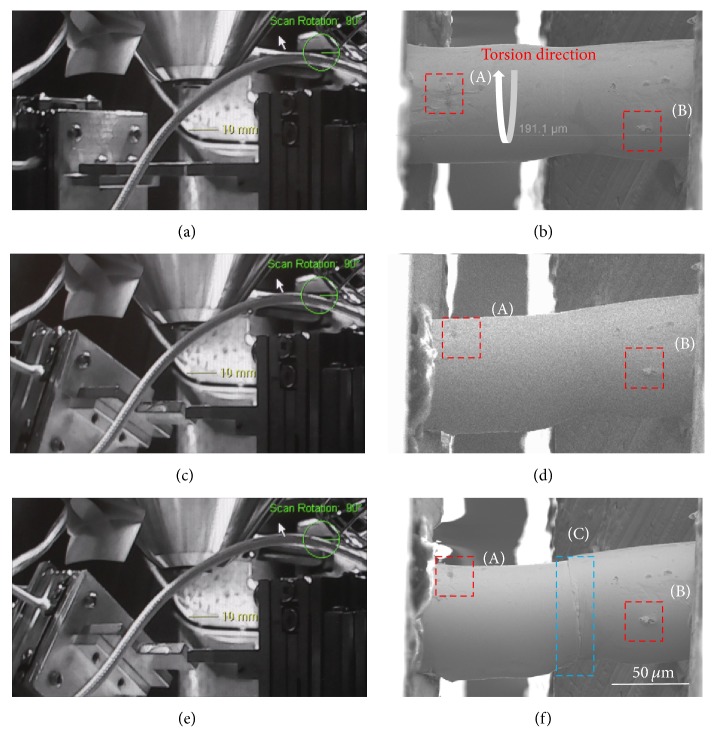
Images selected during the experiment. (a) is the robot pose at the beginning of the experiment. (b) is the sample configuration at the time of (a). The effective length of the sample was about 190 *μ*m. Two markers were selected on the surface to judge whether the rotation happened. The rotation direction was anticlockwise from the left side of view. (c) shows the robot pose during the torsion loading and the displacement of marker (A) was much more obvious than marker (B) as (d) shows. The sample fractured at about 55 degrees of rotation as shown in (e). (f) is the final morphology of the sample, which shows that the marker (A) moved a lot and the fracture happened at the middle part of the sample, partly because of the nonuniformity of the sample diameter or internal defects inside the microwire. The scale bar for the (b), (c), and (d) was 50 *μ*m.

After the alignment and fixation process, we closed the SEM chamber and the robot pose in SEM was horizontal at the beginning as Figure 3(a) shows. The original whole sample configuration is shown in Figure 3(b). There was no preload to the sample. The gauge length was about 190 *μ*m. In order to judge whether the sample was being twisted, we selected two obvious markers (red rectangles A and B) on its surface. Then we twisted the sample through rotary positioner with rotation speed kept unchanged at 5 deg/s and the twisting direction was anticlockwise from the left side of view as shown in Figure 3(b).  Figure 3(c) shows the robot setup during the torsion loading with torsion angle about 45°. From Figure 3(d), captured from the supplementary video (see Supplementary Material available online at https://doi.org/10.1155/2017/6215691), it is easy to find that part of the maker (A) rotated outside of the view and marker (B) almost stayed at the same place. The movement of the markers on the sample can indicate that the clamping was firm enough. Figures 3(e) and 3(f) were images to show the robot pose and sample morphology when fracture happened at 55°.

## 3. Results and Discussions

The shear strain can be calculated by *γ* = *φ∗R*/*L*, in which *φ* indicates the rotation angle; *R* and *L* are the diameter and effective length of the microwire. According to the images captured during the experiment, the MG microwire (dia. 70 *μ*m) with length about 190 *μ*m fractured at about 55 degrees of distortion, which means the maximum shear strain of the sample was about 17.6%. According to the rotation theory, the maximum shear strain located at the rightmost side of the sample between the clamp. However, the fracture that happened at the middle part of the sample may be because of the nonuniformity of the diameter or internal defects inside the microwire.

**Figure 4:**
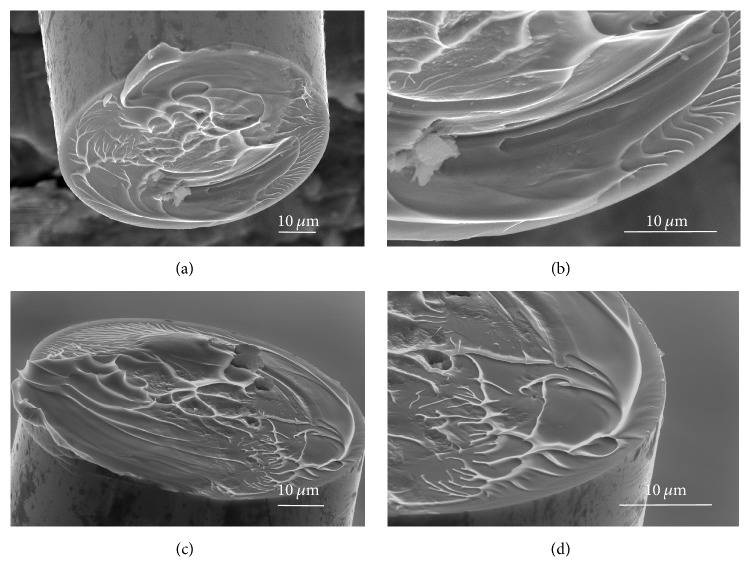
Microstructure of the fracture surface. (a) and (c) are the two corresponding sides at the fracture point. There are revolving vein-patterns on both of them. The magnification images of (b) and (d) show that the area between the corrugations is very flat and clean, which may be because the plastic deformation was confined to extremely small space inside metallic glass.

The overall fracture surfaces of the two sides of MG microwires are shown in Figures 4(a) and 4(c), from which we can easily identify that the vein-pattern microstructures, a typical fracture surface feature of glassy materials, revolved along with the twisting direction. They were different from the microstructure of the fracture surface after tensile loading (as shown in [24]), which means that the fracture was indeed caused by torsion loading. These vein-patterns bear the signature of liquid-like flow occurring inside MG materials. Upon magnification (Figures 4(b) and 4(d)), we also found that there were almost no localized shear bands on the sample fracture surfaces.

At the start period of torque exertion, the plastic deformation was prevented because of lacking structural dislocation, and the stress was usually confined to elastic regime. With the increase of stress, the plastic deformation was usually confined to extremely localized areas (plastic zones) in the material, which caused a rapid temperature rise while the adiabatic heating leads to extremely fast events within a few hundred nanoseconds. The absence of necking in or around the fracture location as Figure 3(f) proved that the plastic deformation was localized at the fracture point. During this time, the material melted and a fluid layer was produced. The local density (as well as the viscosity) of the fluid layer was also changed, and intermixing of two liquids with different densities was responsible for producing such fractal-like patterns, possibly due to Rayleigh-Taylor instability [46].

With the increase of the torsion angle, the nano/microvoid nucleated, and the catastrophic failure happened at the final stage. The rapid cooling of viscous fluid layers leads to the formation of the revolving vein-patterns because of the torsion stress. Obviously, the revolving vein-pattern usually occurred near the edge of the microwire cross-section, as shown in Figure 4, which corresponded to the largest stress at that area. Because the torsion stress decreased towards the center of the microwire, the vein-pattern near it was similar to that of tensile loading.

Compared with the previous mechanical testing of the MG microwires, the presented micro robotic system can speed up the in situ sample alignment process and exert a controllable twist angle on the microwire. The small footprint of the setup is very suitable for in situ SEM mechanical testing, which can give more microstructural information about the fracture mechanism at real time than traditional tests. The robot system utilized the image processing algorithm to ensure the microwire can rotate along the rotary axis and the precise movement of the robot makes it possible to control the effective length of the microwire.

## 4. Conclusion

In this work, in situ SEM torsional tests on the La_50_Al_30_Ni_20_ MG microwires were implemented by self-developed micro robotic mechanical testing system. Firstly, this platform not only reduced the time of alignment involved in microwire torsion test but also increased the precision of it. Secondly, the SEM imaging provided unprecedented details on their fracture state during loading and there was no obvious brittle torsion failure at the cross-section area during the experiment. The vein-pattern microstructure on the fracture cross-section area was very different from that of tensile loading. What is more, the fracture mechanism where the occurrence of the fluid area resulting from adiabatic heating leads to the fracture was revealed. Finally, because of the 6DOFs and precise movement of our platform, it may also be used for micro assembly and micromanufacturing of composite materials at microscale, such as carbon fiber yarns, or mixed protein microfibers [47].

## Supplementary Material

Supplementary Material file contains a supplementary video showing the in situ SEM torsion fracture of a La-based metallic glasss microwire.
